# Applying pharmacokinetic/pharmacodynamic measurements for linezolid in critically ill patients: optimizing efficacy and reducing resistance occurrence

**DOI:** 10.1007/s00228-022-03340-z

**Published:** 2022-05-25

**Authors:** Rasha M. El-Gaml, Noha M. El-Khodary, Rania R. Abozahra, Ayman A. El-Tayar, Soha M. El-Masry

**Affiliations:** 1 Department of Clinical Pharmacy & Pharmacy Practice, Faculty of Pharmacy, Damanhour University, Damanhour City, Egypt; 2 Department of Clinical Pharmacy, Faculty of Pharmacy, Kafrelsheikh University, Kafrelsheikh City, Egypt; 3 Department of Microbiology and Immunology, Faculty of Pharmacy, Damanhour University, Damanhour City, Egypt; 4 Intensive Care Unit, Damanhour National Medical Instititue, Damanhour City, Egypt; 5 Department of Pharmaceutics, Faculty of Pharmacy, Damanhour University, Damanhour City, Egypt

**Keywords:** LZD, Pharmacokinetics, Pharmacodynamics, Continuous infusion, ICU patients

## Abstract

**Purpose:**

Linezolid (LZD) levels are frequently insufficient in intensive care unit (ICU) patients receiving standard dose, which is predictive of a poor prognosis. Alternative dosing regimens are suggested to address these insufficient levels, which are substantial factors contributing to the emergence of multidrug-resistant bacteria, resulting in increased morbidity and mortality among people who are critically ill.

**Methods:**

Forty-eight patients admitted to the intensive care unit were enrolled in an open-label, prospective, randomized study and assigned to one of three LZD administration modes: intermittent groupI (GpI) (600 mg/12 h), continuous infusion groupII (GpII) (1200 mg/24 h) or continuous infusion with loading dose groupIII (GpIII) (on Day 1, 300 mg intravenously plus 900 mg continuous infusion, followed by 1200 mg/24 h on Day 2). We evaluated serum levels of LZD using a validated ultra-performance liquid chromatography (UPLC) technique.

**Results:**

Time spent with a drug concentration more than 85% over the minimum inhibitory concentration (T > MIC) was substantially more common in GpII and III than in GpI (P < 0.01). AUC/MIC values greater than 80 were obtained more frequently with continuous infusion GpIII and GpII than with intermittent infusion GpI, at 62.5%, 37.5% and 25%, respectively (P < 0.01). In GpI, the mortality rate was significantly higher than in the other groups.

**Conclusion:**

In critically ill patients, continuous infusion with a loading dose (GpIII) is obviously superior to continuous infusion without a loading dose (GpII) or intermittent infusion (GpI) for infection therapy. Additionally, it might limit fluctuations in plasma concentrations, which may help overcome LZD resistance.

## Introduction

Linezolid (LZD), the first oxazolidinone, is efficient against Gram-positive bacteria that are both susceptible and resistant to antibiotics, including *methicillin-resistant Staphylococcus aureus (MRSA)*, multidrug-resistant *Streptococcus pneumoniae* and *vancomycin-resistant Enterococci*. This antibiotic is a key therapeutic choice for infections caused by Gram-positive bacterial pathogens that are resistant to other antibiotics in the intensive care unit (ICU). LZD is a medication that has been licensed for the treatment of community-acquired pneumonia, hospital-acquired infection and catheter-related bacteremia in a number of countries [1, 2].

Previous research has shown that LZD improves clinical features and microbiological outcomes in ICU patients. LZD-resistant organisms, on the other hand, have emerged, potentially leading to increased mortality and morbidity in ICU patients [3, 4].

To reduce the risk of underexposure to time-dependent antibiotics in the presence of difficult-to-treat infection, traditional administration has been replaced with a continuous infusion strategy [5]. The combination of pharmacokinetics (PK) and pharmacodynamics (PD) contributes to the optimization of the effectiveness of an antimicrobial agent. In addition, the rational use of PK and PD data enables an understanding of the effect of various dosage regimens on the time course of pharmacologic responses [6, 7]. Optimizing dosing regimens with the help of PK/PD data should also improve patient outcomes and prevent an increase in antimicrobial resistance. The most appropriate LZD’s PK/PD parameters are percentage of time that the drug concentration exceeds the minimum inhibitory concentration (MIC) (%T > MIC), area under the time–concentration curve to MIC (AUC/MIC) and the ratio of the maximum serum antibiotic concentration to MIC (C_max_/MIC) [1].

Patients who are critically ill are frequently at a greater risk of developing multidrug-resistant Gram-positive bacterial infections that are a significant public health concern. The majority of patients admitted to the ICU with bacteremia have significantly higher mortality rates than other patients. To put it another way, incorrect antimicrobial usage is a significant risk factor in the development of multidrug-resistant microorganisms, which increases morbidity and mortality. There was a higher success rate when T > MIC was more than 85% and AUC/MIC values ranged from 80 to 120 in studies of seriously ill adult patients [8, 9].

Our study aimed to compare three different modes of LZD administration in critically ill patients: intermittent (GPI), continuous infusion (GPII) and continuous infusion with a loading dosage (GpIII). In addition, to determine which is better for predicting clinical response and optimizing patient-specific therapy based on the PK/PD profile.

## Subjects and method

This was a prospective, randomized, open-label trial carried out at Damanhour National Medical Institute’s ICU. The procedure was authorized by the Research Ethics Committee of Faculty of Pharmacy, Damanhour University (No. 1021PP40F). All patients provided written informed consent to participate in the study after being informed of the study’s aims and potential risks.

### Subjects

Forty-eight Egyptian patients aged 23 to 87 years, weighing 93.65 kg and having a body mass index (BMI) between 25 and 42 kg/m^2^ were recruited from the ICU. The inclusion criteria included patients who were documented by Gram-positive pathogens sensitive to LZD therapy, fever (> 37.8 °C (100°F) and leukocytosis; white blood cells (WBCs) > 10,500 cells/mm^3^) were considered indicators of infection. The primary attending physician prescribed LZD to treat a bacterial organism that had been collected from a usually sterile place and was compatible with an infectious process.

Patients with any of the following criteria were excluded from participation: pregnancy, experienced a known adverse reaction to LZD in the past, platelet count < 80,000/mm^3^ or had a creatinine clearance (CrCl) < 40 ml/min which was estimated using the Cockcroft–Gault formula [10].

### Study design

Participants were randomly allocated to one of three groups (n = 16) based on the recommended mode of LZD administration. All patients received the same total daily dose of 1200 mg. LZD was administered intravenously (IV) to GpI as a 30-min intermittent IV bolus (600 mg/12 h). LZD (1200 mg/24 h) was administered as a continuous infusion to GpII without previous loading dose administration. On the first day, LZD was given to GpIII as a 300 mg IV loading dosage is followed by a 900 mg continuous IV infusion, followed by a 1200 mg daily continuous infusion.

Medical records of patients assigned to the ICU revealed diagnostic data and data of their treatment plans. The Simplified Acute Physiology II Score (SAPS II) was used to predict mortality rates; WBCs and platelets were also evaluated. All participants in the study got recommended treatment for the infectious illness in accordance with recognized criteria. As clinically required, crystalloids and colloids were utilized to replenish fluids. Microbiological cultures were isolated from blood or any other possible source of infection before the first dosage of LZD.

### Sample collection

Blood samples were taken from each patient treatment using an indwelling IV cannula implanted into the forearm’s antecubital vein; 10 mL was analyzed for complete blood cell count, urea, electrolytes, random blood glucose, liver function tests and renal function tests. Another 5 mL of blood sample was taken from each patient prior to LZD treatment.

The samples were collected in heparinized tubes at 2, 6, 12, 24, 36, 48, 60 and 72 h in patients from all groups (40 ml blood samples in total for each patient). At room temperature, blood samples were centrifuged at 4000 rpm for ten minutes, then plasma was collected over a period of six months and kept at – 80 °C until the LZD concentration was determined (during the time of samples collection) using a validated ultra-performance liquid chromatography (UPLC) technique. Additionally, urine samples for microscopic examination were taken.

### Bioanalytic assessment of LZD

The quantities of LZD in serum were assessed using a validated UPLC technique. The mobile phase was composed of 0.2 percent phosphoric acid and acetonitrile in a 75:25 (vol/vol) ratio. The analysis was performed at a flow rate of 1.5 mL/min using an HPLC column Microsorb-MV 100–3 C18 (250 mm 4.6 mm, 5 mm; Agilent Technologies, the Netherlands) [5, 11, 12]. The effluent was monitored at a wavelength of 254 nm using an ultraviolet (UV) Agilent detector 1290 DAD (Model: G4212A; Serial No. DEBAF04676, USA) and an internal standard (IS; tinidazole). The peak areas of the drug and IS were electronically combined using an analysis program (Agilent Scientific Instruments) and the peak area ratio of the drug to IS was calculated.

The calibration standards for LZD were prepared by transferring 25 μL of each working solution and IS (tinidazole) to a set of test tubes. After evaporating the methanolic solvent, 0.25 mL blank plasma was added to each tube to create a set of calibration standards with concentrations of 1, 2, 5, 10, 20, 50 and 100 mg/L. After vortexing the sample, 0.4 mL acetonitrile was added, mixed for 2 min and then centrifuged for 6 min at 3000 rpm. After separating the supernatant, 20 μL was fed into the UPLC.

0.25 mL of each sample was transferred to a clean test tube for analysis. After adding the IS, the research samples were used as calibration standards (tinidazole). LZD and IS had a mean retention time of 4.33 ± 0.09 min and 3.50 ± 0.07 min, respectively, under the chromatographic conditions stated before.

LZD calibration curve linearity in human plasma was established using least-squares linear regression analysis of the peak area ratios of LZD to IS *vs* the corresponding LZD levels. Over the assay range (1–100 g/mL), the calibration curves were linear, with a correlation coefficient greater than 0.99. Within-day coefficient of variances (CVs) for LZD varied from 1.226 to 5.625%, but between-day CVs ranged from 2.371 to 6.680%.

### PK analysis

LZD’s PK characteristics were determined as follows: AUC values were determined for the periods 0–24 h, 24–48 h and 48–72 h (AUC_0–24_, AUC_24–48_, AUC_48–72_), minimum and maximum concentrations of LZD at steady state (C_pss max_, C_pss min_), the elimination rate constant (K_e_), the half-life (t_1/2_), clearance (CL) and volume of distribution (V_d_). The PK parameters for LZD were determined using a non-compartmental model employing plasma drug concentration–time data. K_e_ for plasma concentration–time data points in the terminal log-linear area of the curves was determined using least-squares regression [7].

T_1/2_ was determined by dividing ln2 by K_e_. The linear trapezoidal rule was used to determine the (AUC _0–t_). The AUC from zero to infinity (AUC_0–∞_) was calculated as AUC_0–∞_ (AUC_0–∞_ + C/K_e_). C denotes the last concentration determined C_max_ and time to C_max_ (i.e., T_max_) were determined directly from the concentration–time curves of individual plasma samples. V_d_ was calculated as D (1–e^−Kt^) divided by [k_e_ t (C_max_–C_min_. e^−Kt^), where D is the dose, k is the elimination rate constant and t is the infusion time. The mean steady-state concentration is calculated as dosage divided by [V_d_. K_e_. DI]. CL value for LZD computed as (K_e_. V_d_) [6].

#### PK/PD model

MICs were established using broth microdilution [13]. The ratio of the maximal serum concentrations to the infecting organism’s MIC was calculated as (C_max_/MIC). The area under the concentration–time curve for a 24-h dosing interval relative to the organism’s minimal inhibitory concentration (AUC_0–24_/MIC) can be calculated quantitatively using the equation [14].

$$A\mathrm{UC}/\mathrm{MIC}=\frac D{Vd\,.\,MIC}.\frac{t0.5}{0.693}.\frac{24}{DI}$$

The amount of time during a dosing interval when the serum concentration is greater than the MIC (%T > MIC) was calculated analytically using the equation %T > MIC = ln [Dose/(Vd*MIC)]*[t_1/2_/0.693]*[100/DI] [15], where V_d_ denotes the apparent volume of distribution in the central compartment (L/kg), k_e_ denotes the elimination rate constant (h^−1^), MIC is the minimal inhibitory concentration (mg/L) for the organism/antibiotic combination and DI denotes the dosing interval (h) [16].

## Statistical analysis

The Minitab Statistical Package version 16 was used to conduct statistical comparisons between three approach treatments using a one-way analysis of variance model (Minitab, State College, Pennsyl Vania) [17]. A P value of ≤ 0.05 was taken as the level of significance.

## Results

### Baseline characteristics and study population

Forty-eight critically ill ICU patients were randomly allocated to one of three equal groups, (age range, 23–80 years; weight range, 70–110 kg; BMI range, 28.3–42.8 kg/m^2^) and all completed the trial. Patients’ demographic information were reported in (Table 1). There were no statistically significant differences in baseline characteristics between the three therapy groups. The average age and BMI of GpI, II, III participants were (56.5 ± 12.4 years, 33.17 ± 3.48 kg/m^2^), (59.11 ± 15.7 years, 32.96 ± 2.05 kg/m^2^) and (56 ± 17.63 years, 34.69 ± 3.31 kg/m^2^), respectively. GpI had a considerably higher mortality rate than the other groups. Eight patients died in GpI, five in GpII and just two in GpIII. The remaining patients experienced clinical success.

**Table 1:** Demographic characteristics and biochemical tests of the patients who completed the study

	GpI^a^ (n = 16)	GpII^b^ (n = 16)	GpIII^c^ (n = 16)	*P*-value
Sex, Male/Female	11 F /5 M	7 F /10 M	11 F /5 M	
Entry Diagnosis				
HAP	5	6	6	
VAP	2	1	4	
Sepsis	2	7	2	
Septic shock	4	3	4	
Postoperative	3		1	
Age, years	56.5 ± 12.4	59.11 ± 15.7	56 ± 17.63	0.916
BMI, kg/m^2^	33.17 ± 3.48	32.96 ± 2.05	34.69 ± 3.31	0.221
Creatinine	1.25 ± 0.74	1.34 ± 0.90	1.32 ± 0.82	0.89
CrCl	139.06 ± 153.97	127.23 ± 113.59	117.58 ± 87.66	0.864
SAPS	29 ± 28.45	33.64 ± 29.06	25.7 ± 21.88	0.732
Fluid Intake	3684.6 ± 1801.7	2871.1 ± 1115.8	2944 ± 1029.69	0.109
ALT	116.06 ± 189.17	67.75 ± 50.13	71.56 ± 40.22	0.427
AST	120.19 ± 164.57	69.44 ± 100.40	90 ± 48.63	0.459
Na	140.63 ± 13.12	142.63 ± 10.92	139.44 ± 7.92	0.706
K	4.73 ± 1.3	4.79 ± ± 1.67	4.13 ± 0.67	0.283
Albumin	2.84 ± 0.38	2.63 ± 0.42	2.86 ± 0.34	0.163
WBC	21.3 ± 12.47	15.7 ± 8.69	22.98 ± 14.39	0.215
Bilirubin	1.19 ± 1.57	0.94 ± 0.2	1.73 ± 2.78	0.469
BUN	20.13 ± 9.09	13 ± 5.72	21.56 ± 8.91	0.009
Platelets	243.69 ± 128.93	228.44 ± 103.14	263.94 ± 82.42	0.642
HCO_3_	19.31 ± 6.33	19.41 ± 7.38	20.81 ± 4.6	0.747

*HAP* hospital-acquired pneumonia, *VAP* ventilator-acquired pneumonia, *BMI* body mass index, *CrCl* creatinine clearance, *SAPS II* Simplified Acute Physiology Score II, *ALT* alanine aminotransferase, *AST* aspartate aminotransferase, *Na* serum sodium, *K* serum potassium, *WBC* white blood cells, *BUN* blood urea nitrogen, *HCO*_*3*_ serum bicarbonate

^a^LZD was administered intermittently to patients in GpI (600 mg/12 h)

^b^LZD was administered as continuous infusion (1200 mg/24 h)

^c^LZD was administered as 300 mg bolus followed by 900 mg infusion in Day 1 then 1200 mg/24 h as continuous infusion

^d^The data are expressed as an absolute number

^e^Data are expressed as mean ± standard deviation

At admission, the most frequently encountered clinical diagnoses were hospital-acquired pneumonia (HAP) (n = 5 in GpI, n = 6 in GpII and n = 6 in GpIII), ventilator-acquired pneumonia (VAP) (n = 2 in GpI, n = 1 in GpII and n = 4 in GpIII), sepsis (n = 2 in GpI, n = 7 in GpII and n = 2 in GpIII) and septic shock (n = 4 in GpI, n = 3 in GpII). The mean length of LZD therapy was ten days (range, 7–15 days) across all groups.

Table 2 shows the location of microorganism isolation as well as the MIC of LZD. *Staphylococci* and *Streptococci* were the most frequently isolated pathogens. LZD’s MIC was 2 mg/L in 54% of isolates and 4 mg/L in 46% of isolates.

**Table 2:** Microbiological results in critically ill infected patients receiving LZD intermittently (GpI), continuously (GpII) or continuously with a loading dose (GpIII), in relation to pathogen source, type and MICs

Loading with continuous infusion GpIII						
Patient	Source	Sample	Pathogen	MIC		Outcome
1	Surgery	Blood	*Staph. Aureus*	4		Improved
2	Lung	Sputum	*Staph. Aureus*	2		Improved
3	Lung	Sputum	*Streptococcus*	4		Improved
4	Lung	Sputum	*Streptococcus*	4		Improved
5	Lung	Sputum	*Streptococcus*	2		Improved
6	Lung	Sputum	*Staph. Aureus*	2		Improved
7	Blood	Blood	*Staph. Aureus*	2		Improved
8	Infected CVP	Blood	*Staph. Aureus*	4		Died
9	Lung	Sputum	*Streptococcus*	2		Died
10	Lung	Sputum	*Streptococcus*	2		Improved
11	Wound	Swab	*Staph. Aureus*	2		Improved
12	Lung	Sputum	*Staph. Aureus*	4		Improved
13	Blood	Blood	*Staph. Aureus*	2		Improved
14	Lung	Sputum	*Streptococcus*	4		Improved
15	Lung	Sputum	*Staph. Aureus*	2		Improved
16	Blood	Blood	*Staph. Aureus*	4		Improved
Continuous infusion GpII						
Patient	Source	Sample	Pathogen		MIC	Outcome
1	Abdomen	Wound swab	*Staph. Aureus*		2	Improved
2	Blood	Blood	*Staph. Aureus*		2	Died
3	Infected CVP	Blood	*Staph. Aureus*		4	Improved
4	Abscess drainage	Blood	*Staph. Aureus*		4	Died
5	Surgical wound	Wound swab	*Staph. Aureus*		2	Died
6	Lung	Sputum	*Streptococcus* + *Staph. Aureus*		2	Improved
7	Lung	Sputum	*Staph. Aureus*		4	Died
8	Lung	Sputum	*Staph. Aureus*		2	Improved
9	Lung	Sputum	*Staph. Aureus*		2	Improved
10	Blood	Blood	*Staph. Aureus*		4	Improved
11	Blood	Blood	*s(MRSA)* + *CONS*		2	Improved
12	Blood	Blood	*Streptococcus*		4	Improved
13	Blood	Blood	*Streptococcus*		4	Improved
14	Lung	Sputum	*CONS*		2	Improved
15	Lung	Sputum	*Streptococcus*		4	Died
16	Lung	Sputum	*Staph. Aureus*		4	Improved
Intermittent GpI						
Patient	Source	Sample	Pathogen	MIC		Outcome
1	Wound	Swab	*Staph. Aureus*	4		Died
2	Blood	Blood	*Staph. Aureus*	2		Improved
3	Lung	Sputum	*Streptococcus*	4		Died
4	Blood	Blood	*CONS*	4		Died
5	Lung	Sputum	*Staph. Aureus*	4		Improved
6	Lung	Sputum	*Staph. Aureus*	2		Improved
7	Lung	Sputum	*Staph. Aureus*	2		Improved
8	Blood	Blood	*Staph. Aureus*	2		Improved
9	Blood	Blood	*MRSA*	2		Died
10	Blood	Blood	*MRSA*	4		Died
11	Wound	Swab	*Staph. Aureus*	4		Improved
12	Blood	Blood	*Staph. Aureus*	4		Improved
13	Blood	Blood	*Staph. Aureus*	2		Died
14	Lung	Sputum	*Staph. Aureus*	2		Died
15	Lung	Sputum	*Staph. Aureus*	2		Improved
16	Blood	Blood	*Staph. Aureus*	2		Died

*MRSA* methicillin-resistant Staphylococcus aureus, *CVP* central venous pressure, *CONS* coagulase negative staphylococcus aureus, *MIC* minimum inhibitory concentration

### Pharmacokinetics

**Fig. 1:**
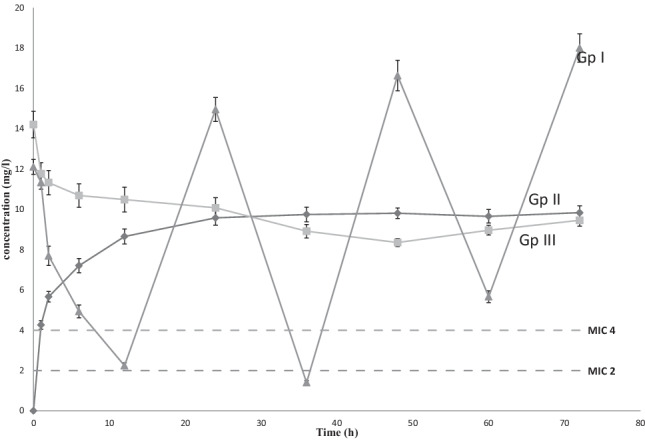
Comparison of the mean serum LZD concentrations in critically ill patients; among 3 groups; intermittent GpI, continuous GpII and loading GpIII after intravenous administration of 1200 mg/24 h through 72 h (n = 16)

LZD’s mean serum concentration–time profiles were measured in the three groups of patients as shown in (Fig. 1). The major PK parameters of LZD were summarized in (Table 3). The serum C_max_ of LZD was 12.1 ± 1.47 mg/L in GpI, with an average blood concentration of 11.33 ± 1.34 mg/L after 0.5 h. Following then, LZD’s mean plasma concentration rapidly decreased from 7.69 ± 1.93 to 4.93 ± 1.28 mg/L, eventually reaching 2.24 ± 0.56 mg/L (at 2, 6 and 12 h, respectively). The plasma concentration of LZD was determined every 12 h for 72 h following administration (Fig. 1). At 1, 2, 6 and 12 h, the mean serum LZD concentrations in GpII were 4.26 ± 0.85, 5.66 ± 1.04, 7.2 ± 1.43 and 8.65 ± 1.49 mg/L, respectively. In GpIII, the mean C_max_ for total LZD was 14.2 ± 2.63 mg/L, with no significant difference from GpI. LZD concentrations at 1, 2, 6 and 12 h of continuous infusion were 11.76 ± 2.22, 11.32 ± 2.42, 10.68 ± 2.33 and 10.48 ± 2.47 mg/L, respectively. At 6 and 12 h, the latter two values were considerably greater than the GpI comparable trough concentrations (P < 0.01). This statistically significant difference in serum LZD concentrations remained constant throughout the experiment. The total trough concentrations after intermittent infusion (GpI) were substantially lower than the mean concentrations reported during continuous IV infusion in GpII and III (Fig. 1). There was no significant difference among the three groups in terms of CL, V_d_, K_e_ or t_1/2_. However, the mean AUC values for LZD over the course of the research (3 days) were significantly different among the three groups (Table 4).

**Table 3:** A single 600 mg intravenous dosage of LZD was administered to eight critically ill patients

Parameter	Mean ± SD
C_max_ (mg/L)	12.1 ± 1.47
K	0.13 ± 0.02
T_½_ (h)	5.53 ± 1.14
AUC_0–12_ (mg h/L)	65.11 ± 12.25
V_d_ (L)	49.01 ± 11.32
CL (L/h)	5.6 ± 1.25

*S.D.* standard deviation, *C*_*max*_ peak serum concentration, *K* elimination rate constant*, t*_*1/2*_ half-life, *AUC*_*0–12 h*_ area under the serum concentration–time curve from 0–12 h, *V*_*d*_ volume of drug distribution, *CL* drug clearance

**Table 4:** Pharmacokinetic/pharmacodynamic parameters of LZD in critically ill patients following intermittent infusion (GpI) or continuous infusion (GpII) of 1200 mg/24 h or after loading dose (GpIII)

Parameter	GpI	GpII	GpIII
AUC_0-24_ (mg h/L)	172.65 ± 28.02^a**^	189.61 ± 30.19^a^	255.31 ± 51.94^a^
AUC_24-48_ (mg h/L)	271.82 ± 46.57^a**^	342.39 ± 43.67^a^	340.7 ± 53.11^a^
AUC_48-72_ (mg h/L)	253.6 ± 46.9^a**^	350.82 ± 42.2^a^	317.81 ± 31.79^a^
T_1/2_ (h)	5.53 ± 1.14	7.44 ± 1.08	4.13 ± 0.37
K	0.13 ± 0.02	0.1 ± 0.02	0.17 ± 0.02
CL (L/h)	5.6 ± 1.25	5.16 ± 0.56	6.19 ± 0.72
Vd (L)	49.01 ± 11.32	55.75 ± 12.18	37.13 ± 7.21
CPss_min_ (mg/L)	4.03 ± 2.07	––	––
CPss_max_ (mg/L)	16.30 ± 4.85	––	––
C_max_ (mg/L)	16.70 ± 3.11	9.65 ± 1.35	8.96 ± 0.96
C_max_/MIC	6.28 ± 1.8	3.55 ± 1.16	3.46 ± 1.09
C_max_/_AVG_MIC	5.81 ± 1.08	3.22 ± 0.44	3.12 ± 0.33
Mean AUC/MIC	64.93 ± 17.03^a**^	68.87 ± 19.9^a^	89.33 ± 21.99^a^
AUC/MIC > 80	4 of 16 (25%)^b^	6 of 16 (37.5%)^b^	10 of 16 (62.5%)^b^
%T > MIC	101.63 ± 26.24	90.36 ± 16.11	61.71 ± 9.17
%T > MIC (2 mg/L)	120.14 ± 19.48	106.09 ± 9.83	69.10 ± 2.86
%T > MIC (4 mg/L)	74.07 ± 13.55	75.06 ± 6.52	51.90 ± 2.10
%T_f_ > MIC	35.42%	100%	100%
%T_*f*_ > MIC (2 mg/L)	77.08%	100%	100%
%T_*f*_ > MIC (4 mg/L)	Zero %	100%	100%

*AUC* area under the serum concentration–time curve, *MIC* minimum inhibitory concentration, *T > MIC* time with serum concentrations higher than the MIC

^*^P < 0.05 GpI versus GpII versus GpIII

^a^Mean ± standard deviation

^b^No. of patients/total patients

### An evaluation of therapy efficacy based on PK and PD data

Following treatment, the likelihood of cure was determined in these patients by analyzing both clinical features and microbiological responses (Table 2). Additionally, the following PK/PD characteristics were included in the analysis as indicators of LZD efficacy: area under the curve (AUC_0–24_/MIC), highest serum antimicrobial level relative to the MIC (C_max_/MIC) and percentage of dosing intervals with a serum concentration higher than the MIC (% T/MIC) (Table 4).

Mean trough levels in GpI were primarily less than the susceptibility breakpoint (4 mg/L), whereas mean trough levels in GpII and GpIII were always greater than the susceptibility breakpoint (4 mg/L). AUC/MIC ratios larger than 80 were obtained in only 25% of all samples (GpI), 37.5% of all samples (GpII) and 62.5% of all samples (GpIII) (P < 0.05), indicating a greater intra-individual variation in GpI than in GpII or III. For GpI, II and III, the C_max_/average MIC values were 5.81, 3.22 and 3.12, respectively. For GpII and GpIII MICs of two mg/L and four mg/L, the mean %T_f_ > MIC was 100% as shown in (Table 4). However, the % T_f_ > MIC in GpI was 77.08% and 0% for two mg/L and four mg/L MICs, respectively. Clinically and microbiologically cured Group II and III patients with an AUC_0–24_/MIC > 80 and T_f_% > MIC is 100% for MIC 2 and 4. By comparing the pharmacodynamics parameters such as AUC/MIC and T > MIC between each two groups using post hoc test, we found there was statistically significant difference between GpI and GpIII and between GpII and GpIII (Table 5). As a result, a statistically and clinically meaningful difference was discovered between GpII and GpIII.

**Table 5:** Post hoc **(**Scheffe) pairwise comparisons method was done to conclude which mean was in difference for comparing between the studied groups

	Group I (*n* = 16)	Group II (*n* = 16)	Group III (*n* = 16)	*p*-value
K				
Min. – Max	0.11 – 0.17	0.08 – 0.18	0.14 – 0.19	< 0.001^*^
Mean ± SD	0.13 ± 0.02	0.10^a^ ± 0.02	0.17^ab^ ± 0.02	
CL				
Min. – Max	4.01 – 7.85	4.29 – 6.29	5.30 – 8.10	0.008^*^
Mean ± SD	5.60 ± 1.25	5.16 ± 0.56	6.19^b^ ± 0.72	
Vd				
Min. – Max	36.45 – 76.75	27.53 – 78.66	27.53 – 78.66	0.192
Mean ± SD	49.01 ± 11.32	55.75 ± 12.18	55.75 ± 12.18	
t 1/2				
Min. – Max	3.72 – 7.41	3.76 – 8.66	3.58 – 4.79	< 0.001^*^
Mean ± SD	5.53 ± 1.14	7.44^a^ ± 1.08	4.13^ab^ ± 0.37	
AUC/MIC				
Min. – Max	46.01 – 94.19	40.97 – 97.81	50.37 – 130.4	0.002^*^
Mean ± SD	64.93 ± 17.03	68.87 ± 19.91	89.34^ab^ ± 20.48	
AUC 0 – 24				
Min. – Max	123.2 – 217.3	119.2 – 234.9	183.4 – 340.0	< 0.001^*^
Mean ± SD	172.7 ± 28.03	189.6 ± 30.19	255.3^ab^ ± 51.94	
AUC 24 – 48				
Min. – Max	192.9 – 338.0	242.8 – 401.8	268.5 – 423.0	< 0.001^*^
Mean ± SD	271.8 ± 46.57	342.4^a^ ± 43.67	340.7^a^ ± 53.11	
AUC 48 – 72				
Min. – Max	179.6 – 314.4	289.8 – 433.9	252.9 – 368.2	< 0.001^*^
Mean ± SD	253.6 ± 46.90	350.8^a^ ± 42.20	317.8^a^ ± 31.79	
T > MIC 2				
Min. – Max	84.80 – 170.1	69.67 – 110.6	64.88 – 75.57	< 0.001^*^
Mean ± SD	120.1 ± 19.48	106.1^a^ ± 9.83	69.10^ab^ ± 2.86	
T > MIC 4				
Min. – Max	53.80 – 110.57	54.00 – 81.45	47.36 – 56.32	< 0.001^*^
Mean ± SD	74.07 ± 13.55	75.06 ± 6.52	51.90^ab^ ± 2.10	
T > MIC				
Min. – Max	53.80 – 141.2	69.67 – 110.6	49.38 – 75.57	< 0.001^*^
Mean ± SD	101.6 ± 26.24	90.36 ± 16.11	61.71^ab^ ± 9.17	
C max/MIC				
Min. – Max	3.94 – 10.16	2.32 – 6.03	2.09 – 5.24	< 0.001^*^
Mean ± SD	6.28 ± 1.80	3.55^a^ ± 1.17	3.46^a^ ± 1.09	
C max				
Min. – Max	11.19 – 20.58	7.09 – 12.67	7.06 – 10.79	< 0.001^*^
Mean ± SD	16.70 ± 3.11	9.65^a^ ± 1.35	8.96^a^ ± 0.96	

Pairwise comparison bet. each 2 groups was done using post hoc test (Scheffe)

*SD* Standard deviation, *p* p value for comparing between the studied groups

^*^Statistically significant at *p* ≤ 0.05

^a^Significant with Group I

^b^Significant with Group II

## Discussion

LZD is the first-line treatment for multidrug-resistant *Staphylococcus aureus* and vancomycin resistant *Enterococcus*. It is regarded as a primary therapy option for critically ill patients [18, 19].

Most studies on PK and PD have been done on healthy volunteers, but a few have been done in patients with specific illnesses [3, 20]. Furthermore, no comparison of the PK/PD markers for LZD in the most critically ill patients using traditional intermittent dosing with continuous infusion with or without loading doses has been made previously.

In critically ill patients who received 600 mg of LZD every 12 h, blood levels varied substantially, with low trough serum concentrations [21, 22]. This necessitates close monitoring of specific PK/PD features when LZD is administered to those patients [21, 23, 24].

In this research, the goal was to determine the most effective method of administering LZD intravenously in critically ill patients based on PK and PD studies. Additionally, A patient’s pathophysiological state is an important factor in influencing the likelihood of recovery.

LZD has a long duration of antibacterial activity due to its prolonged half-life, which is time-dependent. Due to the fact that it is a time-dependent antibiotic, AUC_0–24_/MIC and %T > MIC are frequently employed criteria for assessing its PD efficacy. It is most bactericidal when unbound concentrations of the medication above the bacterial pathogen’s (MIC) (%T_*f*_ > MIC).

LZD was most effective when the %T > MIC was > 85%, or when the AUC/MIC > 100. Additionally, C_max_/MIC is an excellent predictor of bactericidal activity, but because LZD is a time-dependent antibiotic, larger C_max_/MIC ratios may have little effect on the expected clinical outcome [22–25].

Our study shown that continuous infusion was capable of achieving AUC/MIC values between 80 and 120 and %T > MIC > 85%, resulting in a more favorable outcome with a lower mortality rate in those groups compared to intermittent treatment group. Additionally, more patients obtained %T_*f*_ > MIC values greater than 85% when LZD was given as a continuous infusion rather than intermittently.

PK/PD features are significantly altered in critically ill patients. As a result, providing the same dose of LZD to those patients frequently resulted in suboptimal plasma concentrations when compared to healthy participants, owing to the patients’ variations in drug clearance and volume of distribution [21, 23, 24].

LZD’s clearance and volume of distribution were slightly increased in our study when compared to previously published values for healthy individuals [26, 27]. Our findings indicated that continuous administration of LZD significantly reduced the observed substantial fluctuations in plasma levels reported with intermittent dosing of LZD. Thus, continuous administration provided a means of avoiding the poor dose observed in those critically ill patients.

The majority of patients improved clinically and were cured. Among the three treatment groups, there was a significant variation in the proportion of patients who improved, with a higher rate of improvement found in patients receiving LZD by continuous infusion (1200 mg/24 h). There were no adverse events seen in patients receiving LZD via continuous infusion compared to those receiving the same dose via intermittent infusion (600 mg/12 h).

Constant exposure of bacteria or pathogens to antimicrobials at concentrations close to the MIC is typically associated with the development of antimicrobial resistance [9, 28]. Resistance generated can be overcome by obtaining a plasma concentration greater than the MIC. Continuous infusion of LZD with or without a loading dose resulted in plasma concentrations greater than the MIC (2 or 4 mg/L) (%T > MIC = 100%). In comparison, intermittent dosing resulted in decreased plasma LZD concentrations and a failure to maintain above the MIC (T > MIC (4 mg/L) (LZD Breakpoint) = 0%) during the research period. This is another benefit of continuous infusion over intermittent infusion.

Patients with severely impaired renal function did not participate in the study which prevented us from extending the results to severe renal ICU patients, most patients received other antibiotics concomitantly, which may have influenced the clinical outcome, also restriction of PK analysis to the first 600 mg dose. All these conditions considered as the limitations of the study.

Consequently, further studies with ICU patients who have severe renal impairment are recommended to confirm the clinical benefits and safety of LZD’s continuous infusions in these populations.

## Conclusion

LZD can be given continuously with or without a loading dose to treat infections in people who are very critically ill. Continuous infusions have great advantages over intermittent infusions for treating infections in these people. Additionally, continuous infusions might help maintain acceptable serum levels and limit fluctuations in plasma concentrations, which may help overcome LZD resistance, which is common in ICU patients. Continuous infusion with loading dose has a superior positive outcome clinically and statistically than continuous infusion without loading dose.

## Data Availability

All data generated or analyzed during this study are included in this published article.
